# Association of Pre-Implantation Uterine Artery Doppler with Clinical Pregnancy in Assisted Reproductive Technology: A Systematic Review and Meta-Analysis

**DOI:** 10.3390/medicina61061004

**Published:** 2025-05-28

**Authors:** Antonios Siargkas, Areti Faka, Panagiota Kripouri, Evangelos Papanikolaou, Sofoklis Stavros, Ekaterini Domali, Dimos Sioutis, Chrysi Christodoulaki, Apostolos Mamopoulos, Ioannis Tsakiridis, Themistoklis Dagklis

**Affiliations:** 1 3rd Department of Obstetrics and Gynecology, School of Medicine, Faculty of Health Sciences, Aristotle University of Thessaloniki, Agiou Dimitriou, 54124 Thessaloniki, Greece; asiargk@auth.gr (A.S.); aretifaka@auth.gr (A.F.); amamop@auth.gr (A.M.);; 2 3rd Department of Obstetrics and Gynecology, University Hospital “ATTIKON”, Medical School, National and Kapodistrian University of Athens, 12462 Athens, Greecedsioutis@gmail.com (D.S.); christodoulakichr@hotmail.com (C.C.); 3 1st Department of Obstetrics and Gynecology, Alexandra Hospital Athens, 11528 Athens, Greece

**Keywords:** uterine artery Doppler, pulsatility index, clinical pregnancy, assisted reproductive technology, in vitro fertilization, endometrial receptivity

## Abstract

*Background and Objectives:* This meta-analysis aimed to determine whether pre-implantation uterine artery (UtA) Doppler measurements are associated with clinical pregnancy in women undergoing assisted reproductive technologies (ART). *Materials and Methods:* A systematic search of MEDLINE, Scopus, and the Cochrane Library from inception until 25 March 2025 was conducted to identify relevant studies. Additional records were retrieved through grey literature searching and manual reference checks. Eligible publications were observational studies or clinical trials that evaluated UtA Doppler indices prior to embryo transfer in adult women undergoing ART. Studies were required to report on clinical pregnancy rates, while those enrolling participants under 18 years of age, lacking Doppler data, or providing no pregnancy endpoints were excluded. Three reviewers independently assessed study quality using the Newcastle–Ottawa Scale and the Quality in Prognosis Studies tool. Meta-analyses were performed using a random-effects model to calculate mean differences (MDs) with 95% confidence intervals (CIs). Heterogeneity was examined via Cochran’s Q and the I^2^ statistic. Sensitivity analyses excluded studies at high risk of bias. *Results:* In total, 12 studies met the inclusion criteria, including a population of 3317 women. Women who achieved clinical pregnancy had a lower mean UtA pulsatility index (PI) (MD, −0.26; 95% CI, −0.46 to −0.06) and a higher peak systolic velocity (PSV) (MD, 8.59; 95% CI, 2.31 to 14.87) than those who did not conceive. Subgroup analyses showed that UtA PI measured during the menstrual cycle was lower in clinical pregnancy cases (MD, −0.38; 95% CI, −0.66 to −0.10). Measurements on the day of hCG administration or the day before showed a non-significant difference in UtA PI (MD, −0.43; 95% CI, −1.03 to 0.17), while assessments on the day of embryo transfer showed no significant difference between groups (MD, −0.02; 95% CI, −0.28 to 0.24). *Conclusions:* This meta-analysis suggests that lower UtA PI and higher PSV prior to embryo transfer are associated with higher clinical pregnancy rates in ART, particularly when measurements are taken during the menstrual cycle. Although these findings highlight a potential role for uterine hemodynamics in successful clinical pregnancy, UtA Doppler alone may not be a reliable predictor. Future studies should focus on earlier-cycle Doppler measurements and their integration into multifactorial models to improve prognostic accuracy.

## 1. Introduction

Infertility affects approximately 10% of couples in their reproductive years and exhibits an increasing trend due to the postponement of childbearing, thus representing a significant challenge in reproductive medicine [1]. While progress in controlled ovarian hyperstimulation, in vitro fertilization (IVF), and embryo culture has improved the quality and quantity of embryos, pregnancy rates in IVF and embryo transfer (ET) cycles still approximate 40% [2]. This highlights the continued necessity for a more comprehensive understanding of the determinants of successful clinical pregnancy.

A successful pregnancy following assisted reproductive technologies (ART) requires the confluence of several factors, notably a viable embryo, a receptive endometrium and effective interaction between them [3]. Endometrial receptivity is influenced by a multitude of factors, including its thickness, morphological alterations, and hormonal fluctuations occurring endogenously or through exogenous administration [4]. Furthermore, adequate uterine perfusion has been proposed as a contributing factor to endometrial receptivity [5] because sub-optimal flow can thin the endometrium and disrupt the morphological changes that prime it for embryo acceptance [6,7,8]. In early gestation, a vascular bed that cannot rapidly lower resistance, often reflected by elevated uterine-artery Doppler indices, is less likely to sustain a successful clinical pregnancy [7,8]. These pathophysiological links have prompted the search for objective, ultrasound-based surrogates that reflect both the structural and vascular facets of receptivity.

Among the various methodologies for evaluating endometrial receptivity, endometrial thickness, a well-established sonographic parameter, has demonstrated a positive correlation with clinical pregnancy rates in ART and is widely employed [9]. Nevertheless, endometrial thickness as an isolated variable shows limited predictive capacity and does not fully capture uterine receptivity [10]. In contrast, the role of uterine vascularization, while considered equally significant, remains a subject of ongoing debate [11]. Adequate uterine perfusion has long been considered essential for achieving clinical pregnancy and can potentially be assessed through the application of uterine artery (UtA) Doppler [5,11]. Although UtA Pulsatility Index (PI) is routinely utilized during gestation to evaluate the risk of placental insufficiency [12,13], its predictive value prior to ET is less clearly defined [14]. Numerous investigations have explored the association between pre-implantation UtA Doppler parameters and clinical pregnancy; however, the findings have been inconsistent [15,16,17]. These discrepancies may arise from variations in study population sizes, differences in the ART method and protocols followed and the timing of ultrasound assessments relative to ET.

Given these inconsistencies and the complexity inherent in this area of research, this meta-analysis aimed to systematically review and synthesize the available literature investigating the association between UtA Doppler measurements and clinical pregnancy in ART. By consolidating evidence from diverse studies, we aim to clarify the clinical utility of these vascular markers and identify the most appropriate timing for their assessment within the ART cycle.

## 2. Materials and Methods

This systematic review and meta-analysis were conducted and reported in accordance with the Preferred Reporting Items for Systematic reviews and Meta-Analyses (PRISMA) guidelines [18]. The protocol for this review was prospectively registered in Open Science Framework (https://osf.io/rx8hc/, accessed on 15 May 2025). Since this study is based on previously published, aggregated data, it did not require institutional review board approval or patient consent.

### 2.1. Search Strategy

To investigate the association between pre-implantation UtA Doppler measurements and clinical pregnancy rates among pregnancies following ART, a comprehensive literature search was performed. The databases MEDLINE (via PubMed), Scopus, and the Cochrane Central Register of Controlled Trials (CENTRAL) were searched from their inception to 25 March 2025. The search strategy was built around key terms related to UtA Doppler assessment and ART. For PubMed, the specific search terms were: (Uterine Artery OR UtA) AND (Assisted Reproductive Technology OR ART OR In Vitro Fertilization OR IVF OR Embryo Transfer OR Intracytoplasmic Sperm Injection OR ICSI). Similarly, adapted search strings were used for Scopus and CENTRAL.

In addition to database searching, Google Scholar was screened for relevant grey literature. The reference lists of all included studies and relevant review articles were manually examined to identify any additional eligible studies. Only studies published in English were included.

### 2.2. Study Selection Criteria

Eligible studies for inclusion were observational or clinical trials that examined the relationship between UtA Doppler measurements taken before ET. Specifically, eligible studies needed to include UtA Doppler measurements at one of the following specific time points: during the menstrual cycle, before the administration of human chorionic gonadotropin (hCG), or on the day of ET. The target population consisted of women undergoing ART with documented UtA Doppler measurements. Studies were excluded if they involved participants under the age of 18.

### 2.3. Investigated Outcomes

The outcome was the association between UtA Doppler measurements and successful clinical pregnancy, defined as the presence of a visible gestational sac on the ultrasound scan, following an ET. Doppler measurements closest to the time of ET were prioritized for our primary analysis, although earlier assessments were considered if more proximate measurements were unavailable. This approach was pre-planned to reflect current clinical practice, where no standardized timing for UtA Doppler assessment exists, and measurements closest to embryo transfer are generally preferred; including all available time points in the primary analysis allowed us to capture this real-world variability and maximize data utilization. For statistical purposes, the mean value of the bilateral UtA Doppler indices was used. All the available UtA Doppler measurements with sufficient data, defined as raw data by more than three included studies, were analyzed.

### 2.4. Data Extraction

A standardized data extraction form, developed a priori in Microsoft Excel, was used to collect information from each included study. Two reviewers (A.F. and A.S.) independently extracted the data, and any discrepancies were resolved by discussion or, if necessary, by consultation with a third reviewer (I.T.). The following data were extracted:

Study Characteristics: First author, publication year, journal, country, study design, study period.

Population Characteristics: Inclusion/exclusion criteria, type of ART procedure.

Doppler Measurement Details: Timing of Doppler, specific Doppler index used (PI, RI, etc.).

Quantitative Data: Number of clinical pregnancies and the mean bilateral UtA Doppler index values. When a study reported unilateral UtA Doppler indices values without providing a bilateral mean, we calculated the mean of the two unilateral measurements to derive the bilateral UtA Doppler index value.

For overlapping cohorts, we used the most recent and complete publication. When essential data were unavailable, we contacted the corresponding authors to obtain the missing information.

### 2.5. Quality and Risk of Bias Assessment

The methodological quality of each included study was independently assessed by three reviewers (A.F., A.S., and I.T.) using the Newcastle–Ottawa Scale (NOS) for observational studies [19]. The NOS evaluates studies based on three domains: selection of participants, comparability of groups, and ascertainment of outcome. A maximum of nine stars can be awarded, with higher scores indicating higher quality.

The risk of bias in individual studies was assessed using the Quality In Prognosis Studies (QUIPS) tool [20]. Three reviewers (A.F., A.S., and I.T.) independently evaluated each study across six domains: study participation, study attrition, prognostic factor measurement, outcome measurement, study confounding, and statistical analysis and reporting. Each domain was rated as having a low, moderate, or high risk of bias. Discrepancies in quality assessment and risk of bias ratings were resolved by discussion and consensus.

### 2.6. Data Synthesis and Statistical Analysis

Statistical analyses were performed using Review Manager (RevMan 5.4.1) and RStudio (2024.12.1). The UtA PI, resistance index (RI), and peak systolic velocity (PSV) were analyzed as continuous variables. The study group consisted of those women who achieved clinical pregnancy versus those who did not. We examined the mean differences of UtA Doppler measurements using the inverse-variance method with a random-effects model (DerSimonian and Laird) due to anticipated heterogeneity. Heterogeneity between studies was assessed using the Cochrane Q statistic (with a *p*-value < 0.1 considered statistically significant) and the I^2^ statistic, which quantifies the proportion of total variation in effect estimates due to heterogeneity rather than chance. Publication bias was evaluated visually using funnel plots and quantitatively using Egger’s test, if at least 10 studies were included in the meta-analysis for a given outcome.

### 2.7. Sensitivity Analyses

Sensitivity analyses were planned per our protocol to evaluate the robustness of the findings. This involved excluding studies with a high risk of bias in one or more QUIPS domains. Additionally, when at least three studies per outcome provided adjusted measures, such as adjusted odds ratios or risk ratios, sensitivity analyses would be carried out using these adjusted values.

### 2.8. Subgroup Analyses

A subgroup analysis based on the timing of the UtA PI measurement was conducted to investigate the impact of timing in the differences observed between the study and the control group and whether there was a meaningful factor of heterogeneity.

## 3. Results

**Figure 1:**
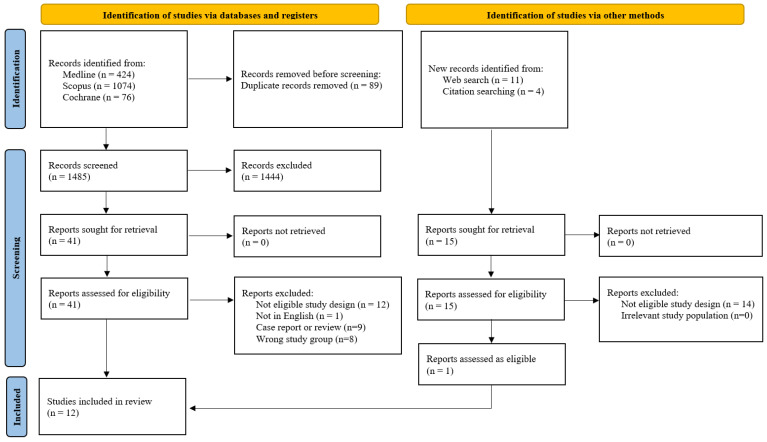
Flowchart of the study selection process.

A comprehensive literature search was conducted on 25 March 2025, across three electronic databases: Medline (n = 424), Scopus (n = 1074), and Cochrane (n = 76), yielding a total of 1574 records. An additional 15 records were identified through web searches (n = 11) and citation tracking (n = 4). After removing 89 duplicates, 1485 unique records were screened. Of these, 1444 were excluded based on title and abstract. Full-text screening was performed for 56 articles in total, and 44 were excluded due to ineligible study design or wrong study group (n = 34), non-English language (n = 1), or being case reports/reviews (n = 9). Five studies that appeared to meet the inclusion criteria were excluded from the analysis for the following reasons: three did not define the outcome of clinical pregnancy [21,22,23], and two defined pregnancy solely based on a positive pregnancy test [15,24], which does not align with our stricter definition (Supplementary Table S1). This resulted in 12 studies being included in the final meta-analysis (Figure 1).

All the included studies are cohort studies [16,17,25,26,27,28,29,30,31,32,33] except one, which is a randomized controlled trial investigating the impact of low-dose aspirin on uterine hemodynamics, but it also offers the relevant raw data for our investigation [34]. Their detailed characteristics are presented in Table 1.

According to the Newcastle–Ottawa Scale assessment (Table 2), most of the included observational studies achieved total scores of 7 or 8, indicating a generally moderate-to-high methodological quality. One study [29] received the maximum of nine stars, reflecting stronger performance across domains of selection, comparability, and outcome assessment. Overall, most studies demonstrated satisfactory quality in participant selection and outcome measures, but a few lacked detailed reporting or proper adjustment for confounders.

### 3.1. Meta-Analyses on the Association Between Uterine Artery Doppler and Clinical Pregnancy

**Figure 2:**
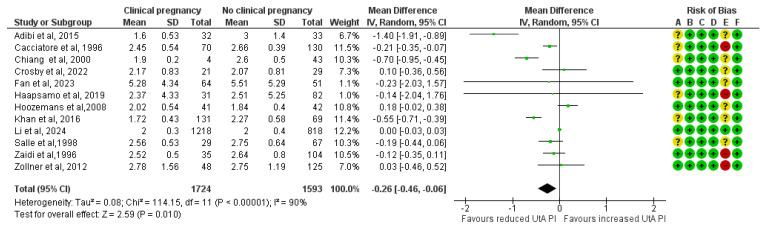
Forest plot comparing the mean uterine artery pulsatility index in those who achieved clinical pregnancies and those who did not [16,17,25,26,27,28,29,30,31,32,33,34]. Abbreviations: CI, confidence interval; IV, inverse variance.

In total, 12 studies assessed UtA PI before implantation among ART pregnancies that achieved a clinical pregnancy (n = 1724) and those that did not (n = 1539). The forest plot suggests that the mean UtA PI before implantation was 0.26 units lower in the clinical pregnancy group, yielding a statistically significant result (MD, −0.26; 95% CI, −0.46 to −0.06; I^2^ = 90%) (Figure 2).

**Figure 3:**
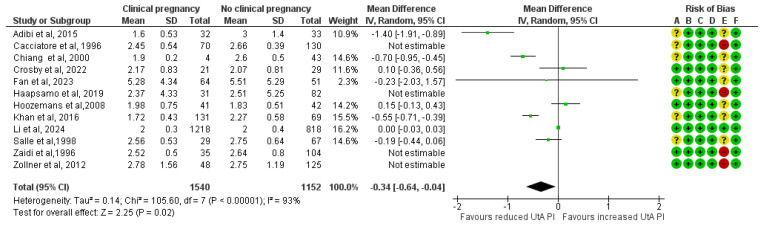
Forest plot comparing the mean uterine artery pulsatility index in those who achieved clinical pregnancies and those who did not, after excluding the high risk of bias studies [16,17,25,26,27,28,29,30,31,32,33,34]. Abbreviations: CI, confidence interval; IV, inverse variance.

The results remained consistent after excluding the four studies classified as high risk of bias. A total of 1540 clinical pregnancies and 1152 non-clinical pregnancies were analyzed, revealing a significant reduction in the mean UtA PI in the clinical pregnancy group (MD, −0.34; 95% CI, −0.64 to −0.04; I^2^ = 93%) (Figure 3).

**Figure 4:**
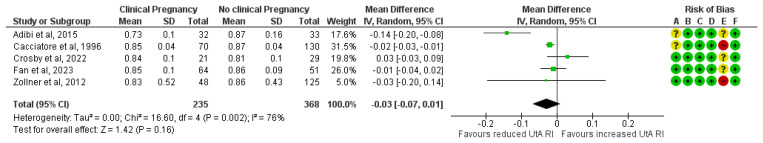
Forest plot comparing the mean uterine artery resistance index in those who achieved clinical pregnancies and those who did not [16,17,25,26,32]. Abbreviations: CI, confidence interval; IV, inverse variance.

Five studies assessed UtA RI among ART pregnancies that achieved a clinical pregnancy (n = 235) and those that did not (n = 368). The forest plot suggests that the mean UtA RI does not statistically significantly differ between the study and the control group (MD, −0.03; 95% CI, −0.07 to 0.01; I^2^ = 76%) (Figure 4).

**Figure 5:**
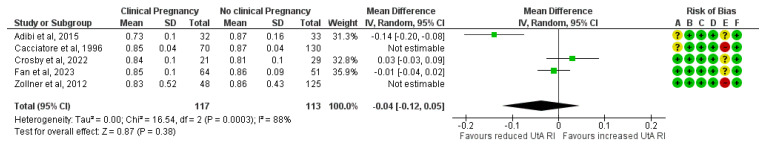
Forest plot comparing the mean uterine artery resistance index in those who achieved clinical pregnancies and those who did not, after excluding the high risk of bias studies [16,17,25,26,32]. Abbreviations: CI, confidence interval; IV, inverse variance.

The association did not reach statistical significance after excluding the two studies classified as high risk of bias. A total of 117 clinical pregnancies and 113 non-clinical pregnancies were analyzed, revealing a non-significant reduction in the mean UtA RI in the clinical pregnancy group (MD, −0.04; 95% CI, −0.12 to 0.05; I^2^ = 88%) (Figure 5).

**Figure 6:**
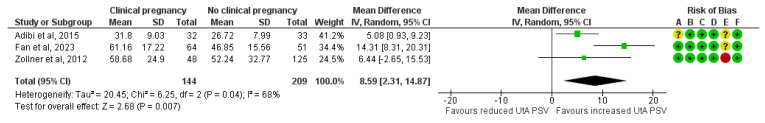
Forest plot comparing the mean uterine artery peak systolic velocity in those who achieved clinical pregnancies and those who did not [16,25,32]. Abbreviations: CI, confidence interval; IV, inverse variance.

Three studies assessed the PSV before implantation in ART pregnancies that resulted in a clinical pregnancy (n = 144) compared to those that did not (n = 209). The forest plot indicates that the mean PSV was 8.6 units higher in the clinical pregnancy group, a difference that was statistically significant (MD, 8.59; 95% CI, 2.31 to 14.87; I^2^ = 68%) (Figure 6). 

**Figure 7:**
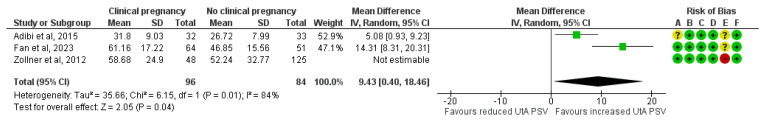
Forest plot comparing the mean uterine artery peak systolic velocity in those who achieved clinical pregnancies and those who did not, after excluding the high risk of bias studies [16,25,32]. Abbreviations: CI, confidence interval; IV, inverse variance.

The results remained consistent after excluding the one study classified as high risk of bias. A total of 96 clinical pregnancies and 84 non-clinical pregnancies were analyzed, revealing a significant increase in the mean UtA PSV in the clinical pregnancy group (MD, 9.43; 95% CI, 0.40 to 18.46; I^2^ = 84%) (Figure 7).

### 3.2. Subgroup Analysis Based on the Timing of the UtA Doppler Measurement

**Figure 8:**
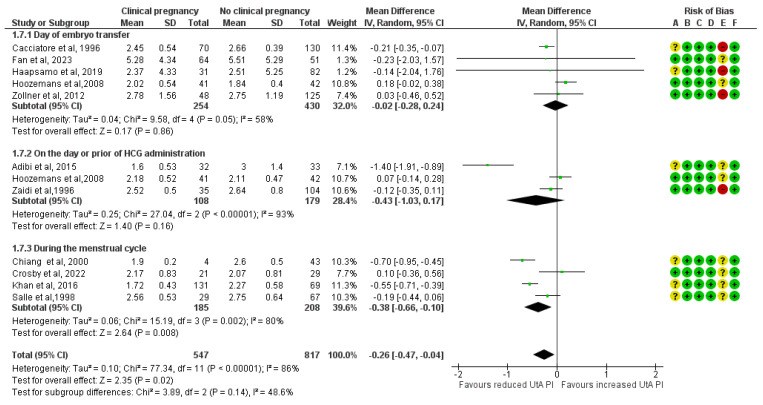
Subgroup analysis based on the time of the mean uterine artery peak pulsatility index measurement comparing those who achieved clinical pregnancies and those who did not [16,17,25,26,27,28,30,31,32,33,34]. Abbreviations: CI, confidence interval; IV, inverse variance.

The UtA PI, when measured on the ET day, has no statistically significant difference between the study and the control group (MD, −0.02; 95% CI, −0.28 to 0.24; I^2^ = 58%). When measured on the day of the hCG administration or the day prior, the mean UtA PI was lower among the clinical pregnancy group, but statistical significance was not reached (MD, −0.43; 95% CI, −1.03 to 0.17; I^2^ = 93%). Additionally, when it was measured during the menstrual cycle the difference was statistically significant and the cases that achieved clinical pregnancies had a lower mean UtA PI by 0.38 units (MD, −0.38; 95% CI, −0.66 to −0.10; I^2^ = 80%) (Figure 8).

### 3.3. Publication Bias

**Figure 9:**
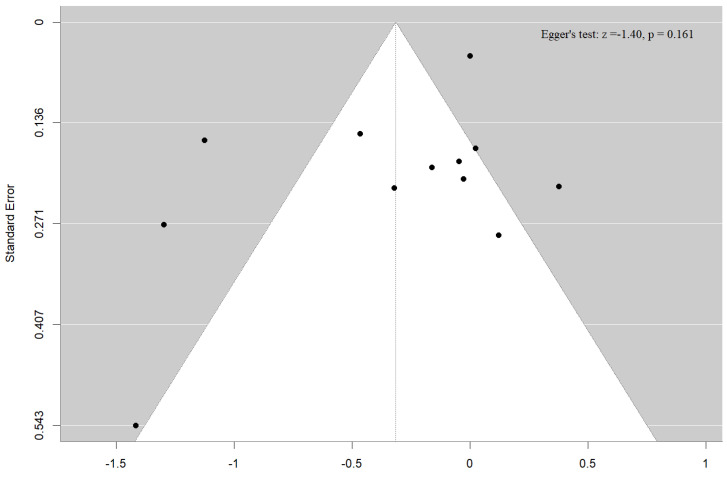
Funnel plot and Egger’s test for the association of uterine artery pulsatility index with clinical pregnancy.

Both the funnel plot and the Egger’s test (*p* = 0.16) failed to detect publication bias for our outcome, the association of UtA PI with clinical pregnancy among ART-conceived pregnancies (Figure 9). Due to the limited number of studies available for the other Doppler indices, reported in fewer than 10 studies, formal publication bias assessments were not performed for these outcomes. This decision aligns with established methodological guidance, which advises against conducting such analyses when study numbers are insufficient to ensure reliable results [35].

## 4. Discussion

### 4.1. Primary Findings

This meta-analysis shows that women who achieve clinical pregnancy after ART have lower UtA PI and higher PSV measured before ET. The difference in PI is clearest when Doppler is performed during the natural menstrual phase, whereas UtA indices obtained on the day of hCG administration or on the ET day are not significantly associated with pregnancy outcome.

### 4.2. Interpretation of the Findings

From a physiological standpoint, the observed association between lower UtA PI and higher PSV in successful ART cycles is biologically plausible. A reduced PI signals diminished downstream vascular resistance and, consequently, increased uterine perfusion, while an elevated PSV reflects the same enhancement in flow velocity [36]. Together, these hemodynamic changes support endometrial thickening, stromal transformation, and trophoblast invasion, processes indispensable for achieving clinical pregnancy [7,8,37]. Although RI showed a similar downward trend in pregnancies, the effect did not reach statistical significance, most likely because only a few studies reported this parameter. Collectively, these Doppler patterns substantiate the concept that uterine blood-flow dynamics are integral to endometrial receptivity and clinical pregnancy.

Uterine receptivity hinges on a finely tuned interaction among a viable embryo, a hormonally synchronized endometrium, and an adequate vascular supply [38,39]. Despite the growing body of research on uterine receptivity markers, a major gap persists in clinically reliable, widely accepted prognostic models. Many current approaches, such as endometrial thickness, morphological grading, or advanced molecular diagnostics like the Endometrial Receptivity Array (ERA), have not definitively translated into better clinical pregnancy rates or consistently predictive outcomes [36,40]. A recent systematic review has emphasized that, while ultrasound-based markers often exhibit high sensitivity, they show poor specificity in predicting pregnancy outcomes and a very poor overall prognostic value [36]. Molecular approaches such as ERA promised a precision-based strategy to personalize ET timing. However, recent studies do not reveal a uniform improvement in live birth rates when ERA is applied to the broad infertility population [40,41]. Consequently, ultrasound markers remain crucial in everyday clinical practice because of their accessibility, noninvasive nature, and cost-effectiveness [42]. Particularly, uterine perfusion is associated with a receptive endometrium through various possible pathophysiological mechanisms. Impaired uterine perfusion can lead to a thinner endometrial lining [37], which has been consistently associated with lower clinical pregnancy rates [6]. Additionally, inadequate perfusion can interfere with essential morphological transformations of the endometrium necessary for receptivity [7,8]. Furthermore, early pregnancy requires rapid and substantial uterine adaptations; thus, a vascular system unable to accommodate these changes, often evidenced by increased resistance in the uterine arteries, is less likely to support a successful clinical pregnancy [7,8].

The role of UtA Doppler has been well established in obstetric settings for forecasting complications such as preeclampsia, fetal growth restriction, and placental insufficiency [14]. Nevertheless, its application before or at the time of ET remains debated; earlier, smaller trials showed inconsistent results, with some reporting no significant link between higher UtA PI and lower pregnancy rates, while others supported a negative association with achieving clinical pregnancy [17,22,23,25]. These discrepancies often stemmed from methodological variations, including limited sample sizes, heterogeneous patient populations, inconsistent timing of measurements, variable ovarian stimulation protocols, and a lack of adjustment for known confounding factors such as maternal age, BMI, and parity, which are recognized to influence uterine artery resistance [43,44]. While gestational age strongly affects UtA-PI, this is unlikely to impact our results since all measurements were taken before implantation [43]. Most studies consistently reported baseline characteristics, showing no major differences between groups; nevertheless, inconsistent adjustment for confounders may have introduced residual bias in the pooled estimates.

One major confounding factor that our subgroup analysis indicated was the timing of the UtA Doppler measurement. The measurements taken earlier in the cycle and particularly during the natural menstrual phase had statistically significant differences between the groups and appear to offer a more physiologically meaningful snapshot of uterine perfusion, likely before any iatrogenic alterations introduced by ovarian stimulation or endometrial preparation [45]. Consequently, these early measurements may better reflect the intrinsic vascular environment that supports endometrial receptivity. In contrast, Doppler assessments performed closer to ET, when the endometrium is under the influence of exogenous hormones, may mask relevant vascular differences and diminish the predictive value of these indices [45]. Clinically, these observations suggest that for UtA Doppler to serve as a meaningful prognostic tool in ART, its assessment should be standardized to the early menstrual phase, before any hormonal or pharmaceutical intervention, to capture baseline uterine vascular conditions most relevant to clinical pregnancy potential.

Our data therefore question the prevailing habit, both in research and in everyday practice, of performing UtA Doppler close to embryo transfer. Measurements acquired earlier in the natural menstrual cycle provide a more representative hemodynamic snapshot of the uterus and should become the standard. Although UtA PI taken on the ET day has limited standalone value, integrating multiple Doppler indices with clinical variables markedly enhances prediction; one retrospective cohort that combined several UtA Doppler parameters with maternal characteristics reported an AUC of 0.782 (95% CI 0.680–0.883) for clinical pregnancy [16]. Building on our findings, future multivariable models ought to include early-cycle UtA PI as a core feature, alongside embryonic morphology [46], genetic screening results, endometrial thickness, hormone profiles, and endocrine immune disorders [47] to generate reproducible, clinically actionable scores that can guide personalized embryo-transfer timing and targeted adjuvant therapies.

### 4.3. Strengths and Limitations

The principal strengths of this meta-analysis include its rigorous and comprehensive methodology. A systematic search was conducted across multiple databases, supplemented by manual reference checks to ensure thorough literature coverage. The inclusion criteria were stringent, with clinical pregnancy clearly defined to exclude studies reporting only positive pregnancy tests and requiring specific timing for UtA Doppler measurements. Furthermore, sensitivity analyses that excluded high-risk studies and conducted subgroup assessments increased the robustness of the results and added significant clinical information.

However, this meta-analysis has several important limitations. While many studies were rated as moderate to high quality, heterogeneity was consistently high across pooled outcomes, likely reflecting underlying variability in study designs, ultrasound measurement protocols, and patient characteristics. The considerable variation in the timing of UtA Doppler assessments across studies significantly restricted the number of studies eligible for inclusion in the time-specific subgroup analysis, limiting the power to draw definitive conclusions for certain time points. Additionally, not all included studies adhered to fully standardized protocols for UtA Doppler measurements. Finally, as discussed in the Interpretation section, the included studies often lacked adjustment for potential confounders, which may have introduced residual bias in the pooled estimates.

## 5. Conclusions

Lower uterine-artery PI and higher PSV measured before embryo transfer are associated with higher clinical pregnancy rates, an effect that is most pronounced when Doppler is obtained early in the ART cycle, ideally during the natural menstrual phase, before any pharmacologic preparation. Standardizing this early-cycle timing across studies and clinical practice could reduce heterogeneity, provide the most physiologically informative hemodynamic snapshot, and sharpen the prognostic value of UtA indices. Although UtA Doppler alone remains an inadequate predictor, early-cycle values integrated with embryonic, endometrial, and maternal factors are well-positioned to strengthen multivariable prediction models and support truly personalized embryo-transfer strategies.

## Figures and Tables

**Table 1:** Characteristics of the included studies.

Study, Year	Study Period	Study Type	Country	Inclusion Criteria	Exclusion Criteria	UtA Doppler Measurement	Time of the Measurements
Adibi et al., 2015 [25]	January 2013 to January 2014	Prospective cohort study	Iran	Age < 40 years, infertile women with frequent abortions, regular menstrual cycle, non-polycystic ovaries at enrollment, follicle stimulating hormone (FSH) < 10 IU/L on day 3.	Tubal factor infertility, history of uterine surgery and/or apparent endometrial pathology, clinically relevant systemic diseases (diabetes, ulcerative colitis, Crohn’s disease, connective tissue diseases, hypertension).	PI, RI, PSV	Day of hCG injection
Cacciatore et al., 1996 [26]	December 1993 to December 1994	Prospective cohort study	Finland	Infertile patients undergoing IVF.	Not reported	PI, RI	Day of ET
Chiang, 2000 [33]	December 1997 to December 1998	Prospective cohort study	Taiwan	Age ≥ 40 years, normal uterine cavity, IVF–ET.	Not reported	PI	During the menstrual cycle
Crosby et al., 2022 [17]	October 2016 to February 2018	Prospective cohort study	Ireland	Age < 38 years, no previous pregnancy, regular menstrual cycles, no steroid hormone use within three months, normal transvaginal ultrasound.	Not reported	PI, RI	During the menstrual cycle
Fan et al., 2023 [16]	April 2021 to September 2021	Retrospective cohort study	China	Age < 38 years, first FET, voluntary uterine artery Doppler monitoring during the implantation window, infertility due to fallopian tube abnormality or mild oligoasthenospermia.	Preimplantation genetic testing, endometriosis or adenomyosis, polycystic ovarian syndrome, ovarian reserve decrease, ovulation disorders, uterine malformations, severe oligospermia/azoospermia/necrozoospermia, ≥2 pregnancy losses, serious medical diseases.	PI, RI, PSV	Day of ET
Haapsamo et al., 2009 [34]	-	Randomized double-blind study	Finland	Less than four previous ovarian stimulations and no contraindications for aspirin.	Not reported	PI	Day of ET
Hoozemans et al., 2008 [27]	March 2002 to December 2003	Prospective cohort study	Netherlands	Age < 39 years, regular menstrual cycle, two normal (non-polycystic) ovaries, FSH < 10 IU/L on day 3.	Tubal factor infertility, history of uterine surgery/endometrial pathology, clinically relevant systemic diseases, smoking, body mass index >28 kg/m^2^.	PI	Day of hCG injection, day of ET
Khan et al., 2016 [28]	June 2011 to April 2014	Prospective cohort study	India	Age 24–43 years, infertility duration 2–20 years.	Patients with secondary infertility and patients with associated male subfertility and infertility	PI	During the menstrual cycle
Li et al., 2024 [29]	October 2019 to September 2020	Retrospective cohort study	China	Age < 40 years, women undergoing FET.	Chromosomal abnormalities, congenital uterine dysplasia, intrauterine adhesions, hydrosalpinx, comorbid medical diseases (hypertension, diabetes, thyroid dysfunction, liver dysfunction, thrombocytopenia), acute hemorrhagic diseases (peptic ulcer), missing cycle data	PI	Day of endometrial transformation before ET
Salle et al., 1998 [30]	1996	Prospective cohort study	France	Women undergoing IVF, normal serum follicle stimulating hormone on day 3 of the cycle	Older than 38 years old	PI	During the menstrual cycle
Zaidi et al., 1996 [31]	1994	Prospective cohort study	London	Infertile women undergoing IVF	Not reported	PI	Day of hCG injection
Zollner et al., 2012 [32]	-	Prospective cohort study	Germany	Couples were included only if at least one fertilized oocyte could be transferred	Women with fibroids of the uterus or with uterine abnormalities were excluded	PI, RI, PSV	Day of ET

Abbreviations: ET, embryo transfer; FET, frozen embryo transfer; FSH, follicle-stimulating hormone; hCG, human chorionic gonadotropin; IVF, in vitro fertilization; PI, pulsatility index; PSV, peak systolic velocity; RI, resistance index; UtA, uterine artery. Quality of the included studies.

**Table 2:** Quality of the included studies.

Study	Study Type	S1	S2	S3	S4	C	O1	O2	O3	Total
Adibi et al., 2015 [25]	Prospective cohort study	b *	a *	a *	a *	a *	b *	a *	a *	8
Cacciatore et al., 1996 [26]	Prospective cohort study	b *	a *	a *	a *	-	b *	a *	a *	7
Chiang, 2000 [33]	Prospective cohort study	b *	a *	a *	a *	a *	b *	a *	a *	8
Crosby et al., 2022 [17]	Prospective cohort study	b *	a *	a *	a *	a *	b *	a *	a *	8
Fan et al., 2023 [16]	Retrospective cohort study	b *	a *	a *	a *	a *	b *	a *	a *	8
Haapsamo et al., 2009 [34]	Randomized double-blind study	b *	a *	a *	a *	-	b *	a *	a *	7
Hoozemans et al., 2008 [27]	Prospective cohort study	b *	a *	a *	a *	a *	b *	a *	a *	8
Khan et al., 2016 [28]	Prospective cohort study	b *	a *	a *	a *	-	b *	a *	a *	7
Li et al., 2024 [29]	Retrospective cohort study	b *	a *	a *	a *	a,b **	b *	a *	a *	9
Salle et al.,1998 [30]	Prospective cohort study	b *	a *	a *	a *	a *	b *	a *	a *	8
Zaidi et al., 1996 [31]	Prospective cohort study	b *	a *	a *	a *	-	b *	a *	a *	7
Zollner et al., 2012 [32]	Prospective cohort study	b *	a *	a *	a *	-	b *	a *	a *	7

Abbreviations: a, first answer according to Newcastle–Ottawa Scale (NOS); b, second answer according to NOS; S, selection; C, comparability; O, outcome; *, attribution of a star according to NOS; **, attribution of two stars according to NOS.

## Data Availability

No original data.

## Supplementary Materials

The following supporting information can be downloaded at: https://www.mdpi.com/article/10.3390/medicina61061004/s1, Table S1. Characteristics of the excluded studies that might appear to meet inclusion criteria according to the PRISMA checklist.

## Abbreviations

The following abbreviations are used in this manuscript:

ART	Assisted Reproductive Technology
BMI	Body Mass Index
CENTRAL	Cochrane Central Register of Controlled Trials
CI	Confidence Interval
ET	Embryo Transfer
hCG	Human Chorionic Gonadotropin
ICSI	Intracytoplasmic Sperm Injection
IVF	In Vitro Fertilization
I^2^	Higgins’ I-squared heterogeneity statistic
MD	Mean Difference
NOS	Newcastle–Ottawa Scale
PI	Pulsatility Index
PRISMA	Preferred Reporting Items for Systematic Reviews and Meta-Analyses
PSV	Peak Systolic Velocity
QUIPS	Quality In Prognosis Studies tool
RI	Resistance Index
UtA	Uterine Artery
