# Supplementary material for: Association of Pre-Implantation Uterine Artery Doppler with Clinical Pregnancy in Assisted Reproductive Technology: A Systematic Review and Meta-Analysis
Source: Medicina (Kaunas). 2025 May 28;61(6):1004. doi: 10.3390/medicina61061004 (PMC12195286; doi:10.3390/medicina61061004)
Supplement: Supplementary file 1 [file medicina-61-01004-s001.zip › medicina-3653279-supplementary.pdf]

Supplementary Table S1. Characteristics of the excluded studies that might appear to meet inclusion criteria according to the PRISMA checklist.

Study, Year	Study period	Study type	Country	Inclusion criteria	Exclusion criteria	UtA Doppler measurement	Time of the measurements	Investigated outcome
Bahrami et al, 2023	January to July 2022	Prospective cohort study	Iran	Age 18–40 years, women candidates for FET.	Severe male factor (azoospermia), egg donation, and uterine surrogacy.	PI, RI, PSV	During the menstrual cycle	Positive pregnancy test
Favre et al, 1993	-	Prospective cohort study	France	Women undergoing IVF with scheduled embryo transfer.	Patients with miscarriage or ectopic pregnancy.	PI	Day of ET	Clinical pregnancy (no definition)
Ozturk et al, 2004	-	Prospective cohort study	UK	Age 24–39 years, women undergoing IVF/ICSI.	-	PI	Day of hCG injection	Clinical pregnancy (no definition)
Prasad et al, 2017	-	Prospective cohort study	India	Age ≤ 38 years, infertile women, baseline (Day 2/3) FSH and LH ≤10 mIU/ml, baseline estradiol ≤50 pg/ml, normal prolactin (<20 ng/ml), TSH <3.5 mIU/ml.	Endocrine abnormalities, previously documented poor response to ovarian stimulation, premature ovarian failure, stage III or IV endometriosis, known psychological disorders, uterine anomalies, uncompensated heart diseases, inadequate endometrial lining (<7 mm).	PI, RI, PSV	During the menstrual cycle and day of hCG injection	Clinical pregnancy (no definition)
Silva Martins et al, 2019	-	Prospective cohort study	Portugal	Women in IVF cycles, with viable good grade embryos for transfer	-	PI, RI	During the menstrual cycle, day of hCG injection and day of ET	Positive pregnancy test

Abbreviations: ET, Embryo transfer; FET, Frozen embryo transfer; FSH, Follicle-stimulating hormone; hCG, Human chorionic gonadotropin; IVF, In vitro fertilization; LH, Luteinizing hormone; PI, Pulsatility index; PSV, Peak systolic velocity; RI, Resistance index; TSH, Thyroid-stimulating hormone; UtA, Uterine artery.
